# Minimally Invasive Transthoracic Device Closure of an Acquired Sinus of Valsalva-Right Ventricle Fistula in a Pediatric Patient

**Published:** 2014-06

**Authors:** Lei Gao, Qin Wu, Xinhua Xu, Tianli Zhao, Yifeng Yang

**Affiliations:** Department of Cardio-thoracic Surgery, The Second Xiang Ya Hospital of Central South University, Xing Ya, China

**Keywords:** Fistula of Sinus of Valsalva; Minimally invasive; Device Closure; Transesophageal Echocardiography

## Abstract

***Background:*** Sinus of Valsalva-right ventricle fistula is a recognized but very rare complication after surgical repair of subaortic ventricular septal defect. Surgical repair with cardiopulmonary bypass and percutaneous transcatheter closure guided by x-ray has been the traditional treatment for fistula of sinus of Valsalva.

***Case Presentation:*** Recently, we have used a novel approach, that avoids the need for either secondary open surgical repair or radiation exposure; that is, minimally invasive transthoracic device closure guided by transesophageal echocardiography to occlude an acquired sinus of Valsalva-right ventricle fistula in a 4-year-old patient.

***Conclusion:*** To our knowledge, there have been no prior cases reported of this technique applied to close an acquired sinus of Valsalva-right ventricle fistula. This report aims to provide a detailed description of the procedure.

## Introduction

Sinus of Valsalva-right ventricle fistula is a recognized but very rare complication after surgical repair of subaortic ventricular septal defect^[^^1^^]^. Cardiopulmonary bypass and percutaneous transcatheter closure are the 2 generally used methods to treat fistula of sinus of Valsalva. Here we present a novel method -minimally invasive transthoracic device closure guided by transesophageal echocardiography (TEE) - that we used to occlude an acquired sinus of Valsalva-right ventricle fistula after surgical repair of ventricular septal defect (VSD) in a 4-year-old patient.

## Case Presentation

**Fig. 1:**
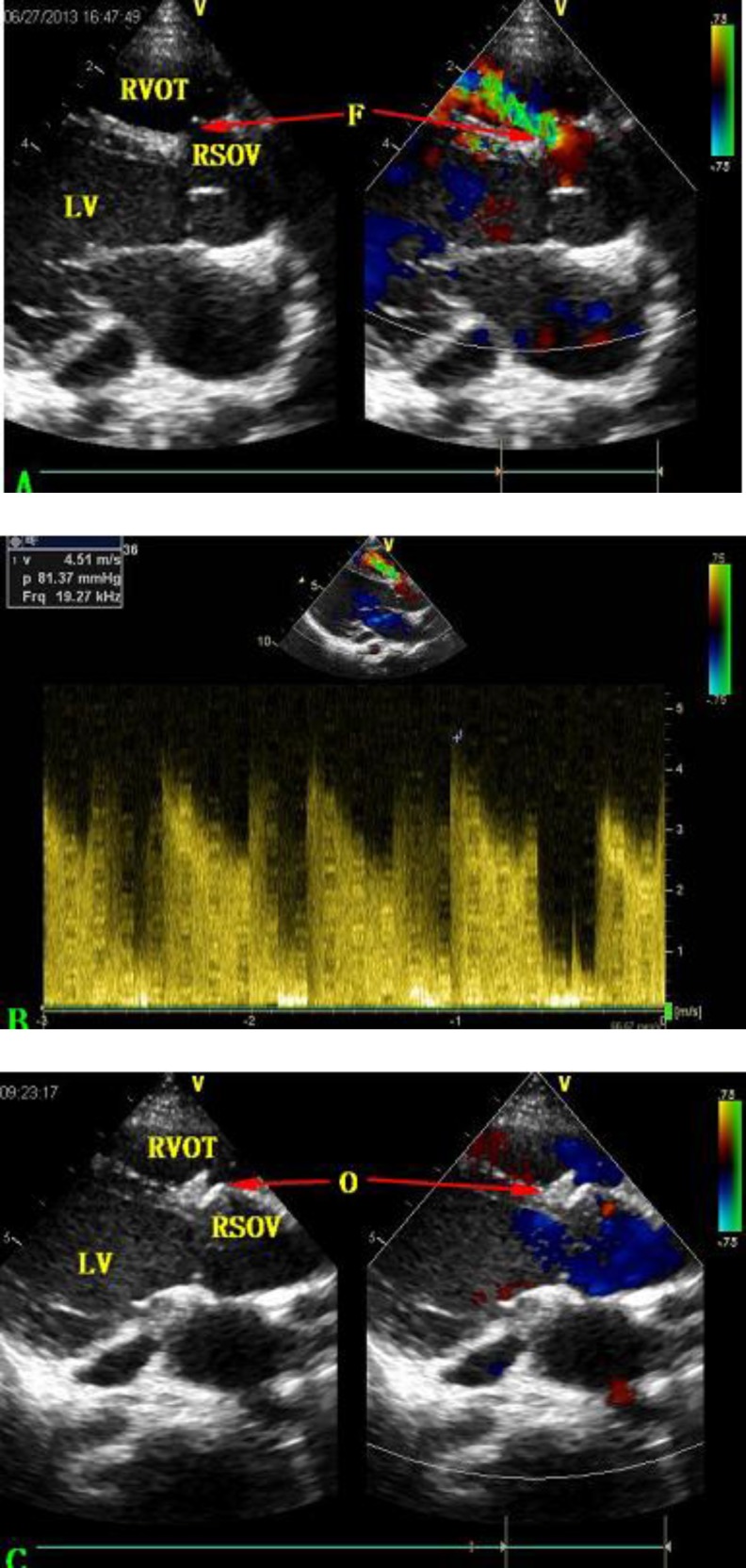
**A:** Transthoracic echocardiography showing the fistula of RSOV to RV. **B:** Color Doppler showing a continuous shunt from RSOV to RV with high velocity (4.5 m/s). **C:** Transthoracic echocardiography revealing satisfactory position of the occluder, with neither residual shunt nor aortic regurgitation

RSOV: Right sinus of Valsalva; F: fistula; RVOT: Right ventricular outflow tract; O: Occluder; LV: Left ventricle; RV: Right ventricle

The 4-year-old girl underwent subaortic VSD repair surgery in our hospital on October 24, 2011 when she was 2 years old. Ten days after surgery, transthoracic echocardiography showed that the VSD was properly repaired but there was a fistula of right sinus of Valsalva (RSOV) to the right ventricle (RV). With the fistula’s diameter of 1.5 mm, color Doppler showed a continuous shunt from RSOV to RV without aortic regurgitation (AR), and physical examination showed a new grade 3/6 continuous murmur at the left sternal border. Since no rupture of sinus of Valsalva was found in the preoperative echocardiography ordetected during surgery, it was believed that this fistula was a postsurgical complication. Because the patient was asymptomatic and the fistula was small, her parents decided to observe her for progression over a period of time. During 21 months of observation, transthoracic echocardiography (once every 3 months) showed that the fistula’s diameter increased from 1.5 mm to 2.5 mm with a high-velocity (4.5 m/s) continuous Doppler flow (Fig. 1A, B, C). Meanwhile, the diastolic diameter of LV also increased from 32 mm to 39 mm. The patient was still asymptomatic and the continuous murmur at the left sternal border became grade 4/6.

 Although the initial treatment recommendation was surgery, the patient’s parents were more inclined to avoid reopening the heart and using cardiopulmonary bypass and eliminating radiation exposure during percutaneous closure. With experience in closed VSDs using minimally invasive transthoracic device closure guided by TEE, we explored the feasibility of adopting this technique to close the fistula. After the potential risks were explained in detail, the parents gave their informed consent for occlusion of the fistula of RSOV under TEE guidance. 

**Fig. 2:**
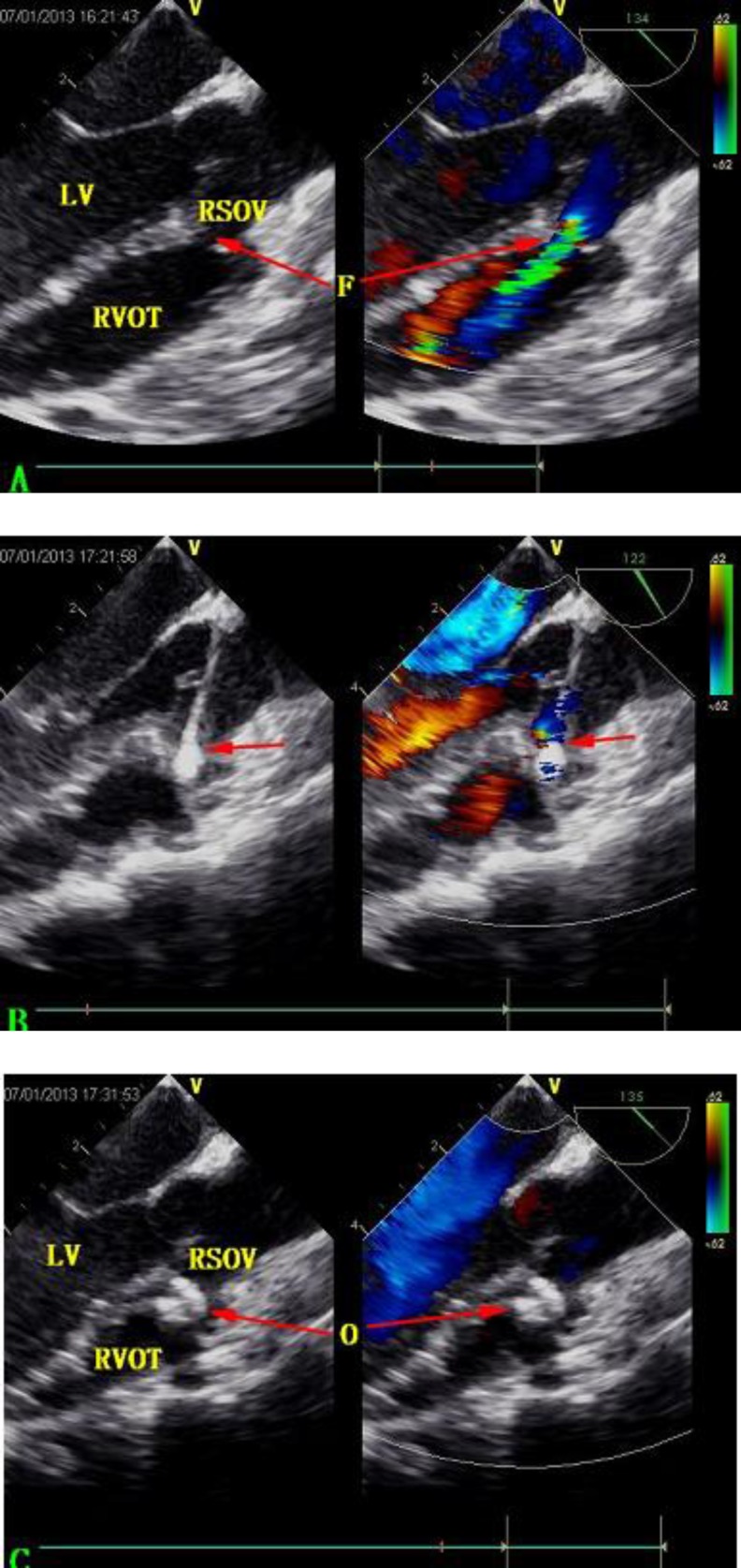
**A** **:** Transesophageal echocardiography showing fistula of RSOV opening into RVOT. **B:** TEE showing the guide wire (arrow) passed through the fistula into RSOV. **C:** Full deployment of the occluder in the fistula after release, with no residual shunt or aortic regurgitation.

RSOV: Right sinus of Valsalva; AoR: Aortic root; F: Fistula; RVOT: Right ventricular outflow tract; O: Occluder; LV: Left ventricle.

 Under general anesthesia, TEE confirmed the diagnosis of fistula of the RSOV to RV (Fig. 2A) with a fistula diameter of 2.5 mm. Antibiotic prophylaxis (Cefotiam, 40 mg/kg body weight) and full heparinization (1 mg/kg body weight) were administered. The free wall of RV was exposed via a 3-cm incision in the inferior sternum and was palpated to locate the area of puncture before a purse-string suture was placed at this location. Under the real-time guidance andmonitoring of TEE (Vivid 7 Dimension, GE, Horten, Norway), a trocar was introduced into RV within the purse-string, and then a guide-wire was passed through the trocar and managed into RSOV through the fistula, at which point the trocar was removed (Fig. 2B). A 7-Fr delivery sheath was fed over the wire and inserted into RSOV through the fistula. With this done, the guide-wire was pulled out, and the previously prepared loading sheath, which contained a 4 mm ordinary symmetric VSD occluder (Shanghai Shape Memory Alloy Corporation, Shanghai, China), was connected to the delivery sheath. The occluder device was then advanced into RSOV through the sheath and thus deployed. 

After deployment, TEE showed that the occluder had a stable position with abolition of the shunt, and no AR occurred (Fig. 2C). No other periprocedural complications were noted. The patient was asked to take 70 mg of aspirin orally daily for 6 months.

 Three months after intervention, the patient was in good condition with no cardiac murmurs on examination.

**Fig. 3:**
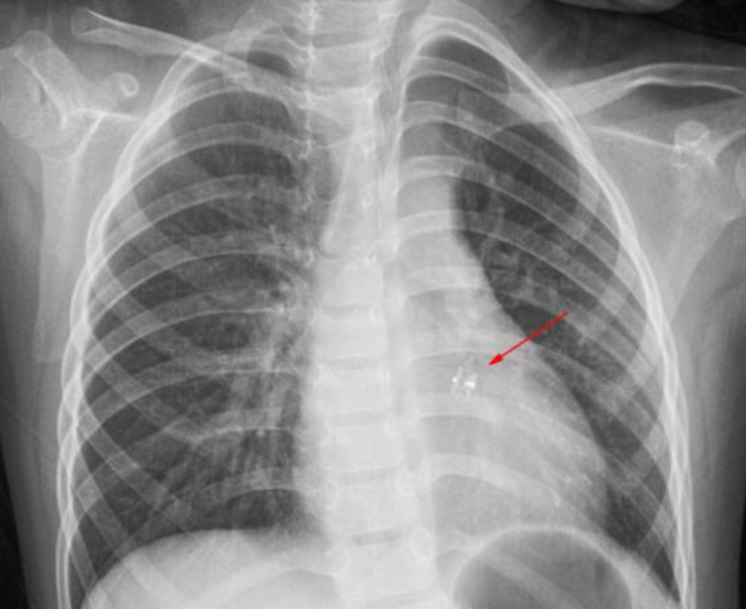
Chest X-ray showing the position of the occluder after occlusion of the sinus of Valsalva-right ventricle fistula (arrow)

Transthoracic echocardiography revealed satisfactory position of the occluder with no residual shunt, aortic regurgitation (Fig. 1C), device embolization or infective endocarditis. The diastolic diameter of LV had decreased from 39 mm to 36 mm. Electrocardiography and chest X-ray also showed normal (Fig. 3).

## Discussion

An acquired Sinus of Valsalva-right ventricle fistula is rare after surgical repair of VSD. Due to the high-pressure gradient across the RSOV to RA, the fistula may cause obvious left-to-right shunt, which will lead to RV overload, or even congestive heart failure. To treat the fistula, repeated open-heart surgery requires a secondary cardiopulmonary bypass^[^^2^^]^, which could increase blood transfusion and cause myocardial dysfunction, especially in a pediatric patient. Since Cullen’s report in 1994, percutaneous transcatheter closure of rupture of the RSOV has been developed^[^^3^^]^. However, performed under x-ray guidance, this procedure involves undesired radiation exposure.

 Minimally invasive transthoracic device closure of fistula of RSOV guided by TEE has a number of potential advantages over open surgical repair. First of all, the need for cardiopulmonary bypass can be avoided, which is especially important in cases where there is hemodynamic instability because of fistula of the RSOV. Secondly, a smaller incision can be used compared with the sternotomy of open-heart surgery. Thirdly, transcatheter device closure may be an alternative method but is limited by the radiation exposure in a pediatric patient. It may also potentiate multiple episodes of tachyarrhythmia and subsequent hemodynamic instability during the procedure. Compared with traditional percutaneous closure, TEE guidance entails no radiation damage^[^^4^^]^. In this procedure, TEE was of utmost importance for (1) sizing the fistula and thus choosing a proper occluder (1–3 mm larger than fistula); (2) understanding the RSOV anatomy with regard to its neighboring structures, namely the aortic valve and tricuspid valve^[^^5^^]^; and (3) most importantly, monitoring AR occurrence and residual shunting.

 Though this approach allows direct manipulation of the device to prevent interference of the valve apparatus,** t**his technique can cause AR because the occluder lies in the sinus of Valsalva; therefore, in case of any serious consequences such as failure of occlusion, obvious aortic insufficiency, or residual shunt, it is indispensable to perform open-heart surgery immediately to avoid any possible danger.

## Conclusion

TEE-guided minimally invasive transthoracic device closure of acquired fistula of RSOV offers a novel alternative to classical surgical repair and percutaneous device closure, since this is a new procedure, appropriate long-term follow-up and management will need to be established.

## References

[1] Matsushita T, Masuda S, Inoue T (2012). Perforation of sinus Valsalva 10 years after repair of ventricular septal defect. Asian Cardiovasc Thorac Ann.

[2] Sarikaya S, Adademir T, Elibol A (2013). Surgery for ruptured sinus of Valsalva aneurysm: 25-year experience with 55 patients. Eur J Cardiothorac Surg.

[3] Cullen S, Somerville J, Redington A (1994). Transcatheter closure of ruptured aneurysm of sinus of valsalva. Br Heart J.

[4] Khoury A, Khatib I, Halabi M (2010). Transcatheter closure of ruptured sinus of Valsalva aneurysm. Catheter Cardiovasc Interv.

[5] Khoury A, Khatib I, Halabi M (2010). Transcatheter closure of ruptured right-coronary aortic sinus fistula to right ventricle. Ann Pediatr Cardiol.

